# Evaluation of the efficacy of mesenchymal stem cells derived conditioned medium in the treatment of striae distensae: a double blind randomized clinical trial

**DOI:** 10.1186/s13287-024-03675-7

**Published:** 2024-03-05

**Authors:** Elham Behrangi, Masoomeh Feizollahi, Sona Zare, Azadeh Goodarzi, Mohammad Reza Ghasemi, Afsaneh Sadeghzadeh-Bazargan, Abbas Dehghani, Maryam Nouri, Roya Zeinali, Masoomeh Roohaninasab, Mohammad Ali Nilforoushzadeh

**Affiliations:** 1 Department of Dermatology, Rasool Akram Medical Complex Clinical Research Development Center (RCRDC), School of Medicine, Iran University of Medical Sciences (IUMS), Tehran, Iran; 2 Skin and Stem Cell Research Center, Tehran University of Medical Sciences, Tehran, Iran; 3 Laser Application in Medical Sciences Research Center, Shahid Beheshti University of Medical Sciences, Tehran, Iran; 4 Stem Cell and Regenerative Medicine Institute, Sharif University of Technology, Tehran, Iran; 5 Department of Mechanical Engineering, Sharif University of Technology, Tehran, Iran; 6 Skin Repair Research Center, Jordan Dermatology and Hair Transplantation Center, Tehran, Iran

**Keywords:** Striae, Striae distensae, Conditioned medium, Microneedling, Efficacy, Clinical trial

## Abstract

**Background:**

Striae distensae is a disfiguring atrophic skin condition that impairs the body’s aesthetic image. Despite the variety of conducted studies, there is controversy regarding the best modalities. Human mesenchymal stem cells are considered a rich source for scar treatment. Skin needling is among the most efficient and safe aesthetic and therapeutic devices. This study aimed to evaluate the efficacy of the combination of needling and intradermal injection of mesenchymal stem cells compared to skin needling alone for treating striae distensae.

**Method:**

This study was a randomized, double-blind clinical trial involving 10 women aged 18–60. Each striae lesion was divided into two parts, with one side receiving needling and intradermal injection of conditioned medium, while the other side received needling and intradermal injection of normal saline. This treatment was administered in three sessions with three-week intervals. Patients were evaluated before the first intervention and three months after the final session. Three months after the completion of the intervention, patients’ lesions were evaluated using biometric criteria, physician evaluation, and patient self-assessment.

**Results:**

The results demonstrated a significant improvement in dermal and complete thickness and skin density in patients treated with microneedling. All skin ultrasound parameters improved significantly in patients receiving the combination of needling and conditioned medium. When comparing the two groups, significantly higher physician and patient satisfaction was observed in the combination group. However, the comparison of biometric indices improvement wasn’t significant between these groups.

**Conclusion:**

The combination of human mesenchymal stem cells with microneedling could be considered a novel effective option for stretch marks.

## Introduction

Stretch marks, scientifically known as striae distensae (SD), are described as disfiguring linear scars, presenting a challenging condition to treat [1]. This relatively widespread condition is more prevalent in women than in men and can affect various age groups [2]. Two forms exist: striae rubrae, appearing as red and swollen scars in the acute stage, and striae albae, characterized by pale, wrinkled, and atrophic white scars in the chronic stage in which end-stage fibrosis occurs [3].

Common sites of skin involvement include the abdomen, hips, thighs, and breasts. Similar to atrophic scars, epidermal and dermal atrophy, as well as alterations in extracellular matrix collagen bundles and elastin fibers, are observed in SD [4]. Stretch marks often develop during pregnancy (striae gravidarum), puberty growth spurts, or rapid body changes such as obesity or weight loss [2, 3]. They can also be associated with medical disorders like Cushing syndrome and genetic disorders such as Marfan syndrome [5]. Certain medications, including prolonged exposure to corticosteroids and antiretroviral protease inhibitors, may contribute to their occurrence [5].

Although these permanent stretch marks are not medically threatening, the increasing demand for effective therapeutic options might be due to their significant impact on psychosocial burden and quality of life [6]. There are quite a number of clinical trials and systematic reviews assessing the effectiveness of different topical treatments and interventions, including lasers, platelet-rich plasma (PRP), radiofrequency, microneedling, dermabrasion, and fillers for treating skin stretch marks [7–10]. Despite the variety of treatment modalities, no specific gold standard has been discovered [8]. Several studies indicated that radiofrequency needling (RF) and lasers have been points of interest for this condition [11, 12]. Among lasers, fractional CO_2_ laser has shown superiority over other laser modalities [12]. The best therapeutic approach to striae was observed in combined treatment methods [7, 8, 11–13]. While topical medication has been widely applied to striae, no specific topical formulation has been shown to be most effective in eradicating or improving SD [13]. Despite the publication of various studies to find the best approach to striae management, there is a discrepancy regarding a highly efficient method for the treatment of SD.

Mesenchymal stem cells (MSCs) were first identified in the 1960s and have since been extensively studied for their unique properties in tissue engineering and regenerative medicine, overcoming many of the limitations of traditional techniques [14]. These cells are a type of stem cell found in various tissues of the body, including bone marrow, adipose tissue, umbilical cord, and amniotic fluid [15]. MSCs have shown promise in treating scars, especially acne scars, wound healing, and skin aging, owing to their unique properties and regenerative capacity [14, 16, 17]. Firstly, MSCs enhance the survival and migration of fibroblasts, cells that produce collagen, and regulate Extracellular Matrix (ECM) remodeling. Production and organization of collagen and elastin in the dermis are essential components of the skin’s extracellular matrix for enhancing the healing process. Secondly, MSCs exhibit paracrine beneficial effects, regulating inflammation and inhibiting myofibroblast differentiation [18, 19]. Additionally, they promote vascular formation that is crucial for proper healing. Finally, their immunomodulatory properties make them a great therapeutic option [20].

The future of MSC research looks promising; however, further clinical trials are substantially needed in this field to validate these theories and animal studies in different conditions, including striae distensae. High proliferation rates, multipotency, hypo-immunogenicity, superior immunomodulation, safety, and better endothelial differentiation potential make Wharton’s jelly mesenchymal stem cells (hWJMSCs) superior to other types of mesenchymal stem cells [21–24]. Additionally, their easy accessibility through non-procedural methods, without ethical concerns, makes them an even better option [25, 26].

Microneedling is a dermatological procedure that involves creating micro-injuries in the skin using fine needles [27, 28]. This stimulates the skin’s natural healing process and promotes the production of collagen and elastin. Several studies have demonstrated the efficacy of microneedling in treating striae distensae [29]. The technique has been found to be safe and effective for both light and dark skin types, with no significant risk of post-inflammatory pigmentation [30].

Given the lack of a gold standard treatment for striae distensae and limited research on the efficacy of MSC-CM in this context, our study aims to explore the impact of hWJMSCs on stretch marks through a blinded randomized clinical trial.

## Materials and methods

### Patient selection

Ten women aged between 18 and 60 years with striae distensae on the abdomen were included in this randomized, double-blind clinical trial. They were referred to a dermatology clinic between February and July 2022. Exclusion criteria included prior treatment for their lesions within the past six months, use of steroids or immunosuppressive drugs, collagen vascular diseases, pregnancy, lactation, the presence of skin infections at the treatment site, and a history of malignancy. All patients provided written informed consent before the initiation of the procedure, ensuring that they understood the therapeutic procedure. Demographic data were collected and recorded on the study checklist.

All ten participants exhibited stretch marks on their abdominal skin. After the initial assessment, one lesion was chosen for each patient. All of the selected stretch marks were identified as striae alba. Each striae lesion was divided into two equal parts. Through random allocation, one side was assigned to receive microneedling and intradermal injection of MSC-CM (MN plus CM Group), while the other side received microneedling and intradermal injection of normal saline (MN plus NS group) as a control group. The baseline condition of SD was assessed both qualitatively through clinical imaging and quantitatively through biometric parameters [31].

### Randomization and blinding

Lesions were randomly allocated to receive either MN plus CM or MN plus NS using a randomized list. The study was conducted as a double-blind trial, with injections administered using identical syringes. Patients remained unaware of the type of treatment they received. Additionally, both evaluating physician and the statistical expert were blinded to each patient’s treatment method.

### Isolation of mesenchymal stem cell from umbilical cord Wharton’s jelly tissue and preparation of conditioned medium

Isolation and culture phases were performed under sterile conditions according to Clinical Research Practice (GCP) instructions in the cell culture laboratory. Longitudinal pieces of the umbilical cords, which were separated from three newborns, were collected in a sterile container and rinsed thoroughly with phosphate-buffered saline (PBS) (Miltenyi Biotec, Cologne, Germany) to remove any blood. Ten milliliters of PBS were supplemented with amphotericin B (1%, Gibco, UK) and penicillin/streptomycin (PS, final concentration of 50–100 I.U./mL penicillin and 50–100 (μg/mL) streptomycin, Gibco, UK) was added to the tube and transported on ice to the laboratory for further processing within 2 h of harvesting the cord.

Under a clean and sterile laminar flow hood, the umbilical cord was washed with PBS and cut into small segments. Sample was then exposed to 0.1% collagenase II (Worthington, USA) for 45 min with intermittent shaking every 5 min in an incubator at 37 °C. To inactivate the enzyme, Dulbecco’s Modified Eagle Medium (DMEM) (Gibco, UK) containing 10% Fetal Bovine Serum (FBS) (Atocel, Austria) was added to the solution. Again, the solution was centrifuged for 10 min at 1500 RPM and the supernatant was removed. Then, the precipitated residue was added to 25 mL of normal saline and was filtered through a 70-µm cell strainer. The cells containing normal saline solution was then centrifuged for 5 min at 1500 RPM. Finally, cells were cultured in 10% FBS and incubated. When the confluence surpassed 80%, cell passage was performed. The aliquoted cells were then frozen as a cell bank for future use.

Cell culture solution from the 3rd to the 5th passage was collected and filtered to serve as CM, and the risk of possible contamination with virus (HIV, Hepatitis A, B, and C,…), bacteria, mycoplasma, yeast, fungi, and endotoxins was evaluated.

### Intervention methods

Thirty minutes before the initiation of the intervention, 2.5% lidocaine/prilocaine cream (Xyla-P, Tehran Chemie Pharmaceuticals, Iran) was applied for local analgesia under occlusion. Before the intervention began, the lesion was cleaned with sterile gauze and disinfected using 70% alcohol swabs.

Microneedling (AMIEA MED microneedling, MT. Derm, Germany) was administered on the entire lesion using single-use cartridges with six sterile needles. Needling was conducted while gently tractioning the lesion, with depths ranging from 1.5 to 2 mm. Multiple horizontal, vertical, and oblique passes were performed until pinpoint bleeding occurred. On the MN plus NS side, intradermal injection of 2–3 units of normal saline serum was performed at one-centimeter intervals using a 1 cc syringe. This technique was also applied on the MN plus CM side using syringes containing hWJMSCs. These interventions were conducted in three sessions with three-week intervals.

A layer of topical antibiotic was applied over the treated lesions without a dresser. Patients were advised to apply the topical antibiotic for three days, avoid sun exposure for several days, and apply zinc oxide as a sunscreen and moisturizer whenever needed.

### Assessment methods

Each patient was examined before the initiation of intervention and three months after the final session, employing the following measures:

1. Biometric assessment: The thickness and density of epidermis, dermis, and the whole skin were measured using ultrasound imaging with a Skin scanner (75 MHz probes, DUB-USB75, TPM, Germany). Additionally, in order to assess tissue elasticity, a cutometer (dual MPA 580, Courage + Khazaka, cologne, Germany) was utilized using parameters such as R2: viscoelasticity, R5: pure elasticity, and R7: proportion of immediate recovery compared to amplitude after suction)
2. Determination of physician and patient satisfaction in both groups was categorized as No Response (0), Little (1), Somewhat (2), Good (3), and Excellent (4). The physician assessor was blind to the study.

### Statistical analysis

Descriptive results were reported as mean (± standard deviation, SD). The paired sample t-test was employed to compare continuous variables before and after the intervention. The analysis of covariance (ANCOVA) test was used to compare biometric factors between the two groups. Fisher’s exact test was utilized for categorical variables. The significance level was set at 0.05. All data were analyzed using SPSS (IBM Corp., Armonk, NY, USA, released 2015).

## Results

A total of 10 females aged 18–60 years with abdominal striae participated in this study. All of them completed it. The average age of the enrolled patients was 34.60 ± 7.03 years.

### Biometric assessment over time

In the MN plus NS group, the measured skin ultrasound indices, including complete thickness, dermal thickness, epidermal density, dermal density, and complete density, improved significantly (*p*-value < 0.05) three months after the intervention. Neither epidermal thickness nor cutometer measurements changed significantly (*p*-value > 0.05). More information is presented in Table 1.

**Table 1:** Comparing the average of biometric factors before and after the intervention in two groups

Group	Index	Mean ± SD (Baseline)	Mean ± SD (3th month)	*p*
Microneedling + conditioned medium	Cutometer R2	0.83 ± 0.09	0.86 ± 0.09	0.37
	Cutometer R5	0.97 ± 0.22	0.89 ± 0.10	0.37
	Cutometer R7	0.61 ± 0.14	0.62 ± 0.11	0.91
	Complete thickness	1099.90 ± 167.59	885.10 ± 110.06	**0.001**
	Epidermal thickness	94.80 ± 8.51	86.40 ± 9.61	**0.002**
	Dermal thickness	1005.10 ± 162.85	798.70 ± 110.39	**0.002**
	Complete density	22.95 ± 4.78	18.98 ± 4.69	**0.006**
	Epidermal density	51.17 ± 4.55	45.53 ± 5.08	**0.005**
	Dermal density	20.21 ± 4.99	16.08 ± 4.95	**0.008**
Microneedling + normal saline	Cutometer R2	0.83 ± 0.09	0.85 ± 0.08	0.47
	Cutometer R5	1.01 ± 0.25	0.92 ± 0.12	0.33
	Cutometer R7	0.58 ± 0.14	0.60 ± 0.12	0.69
	Complete thickness	989.00 ± 131.70	880.30 ± 138.84	**0.003**
	Epidermal thickness	97.90 ± 14.21	88.40 ± 8.80	0.07
	Dermal thickness	891.10 ± 121.52	791.90 ± 136.09	**0.004**
	Complete density	23.23 ± 4.96	21.65 ± 5.84	**0.04**
	Epidermal density	50.69 ± 3.74	47.50 ± 4.40	**0.007**
	Dermal density	20.16 ± 5.08	18.72 ± 5.94	**0.04**

Bold values indicate significant difference for* p*-value

When comparing the biometric parameters between the two groups, both ultrasound and cutometer assessments did not reveal a significant difference at baseline (*p*-value > 0.05) (Table 2). According to the results of the ANCOVA test shown in Table 2, the comparison of biometric parameter changes after three months of follow-up, both ultrasound and cutometer measures between the two groups, did not demonstrate statistical significance.

**Table 2:** Comparing the average of biometric factors between two groups

Index	Mean ± SD	*p*	F	Partial Eta squared
*Cutometer R2*				
Microneedling + conditioned medium	0.83 ± 0.09	0.97	0.001	0.001
Microneedling + normal saline	0.83 ± 0.09			
*Cutometer R5*				
Microneedling + conditioned medium	0.97 ± 0.22	0.67	0.17	0.02
Microneedling + normal saline	1.01 ± 0.25			
*Cutometer R7*				
Microneedling + conditioned medium	0.61 ± 0.14	0.67	0.17	0.02
Microneedling + normal saline	0.58 ± 0.14			
*Complete thickness*				
Microneedling + conditioned medium	1099.90 ± 167.59	0.06	3.94	0.18
Microneedling + normal saline	989.00 ± 131.70			
*Epidermal thickness*				
Microneedling + conditioned medium	94.80 ± 8.51	0.68	0.17	0.02
Microneedling + normal saline	97.90 ± 14.21			
*Dermal thickness*				
Microneedling + conditioned medium	1005.10 ± 162.85	0.05	4.24	0.20
Microneedling + normal saline	891.10 ± 121.52			
*Complete density*				
Microneedling + conditioned medium	22.95 ± 4.78	0.16	2.11	0.12
Microneedling + normal saline	23.23 ± 4.96			
*Epidermal density*				
Microneedling + conditioned medium	51.17 ± 4.55	0.35	0.92	0.06
Microneedling + normal saline	50.69 ± 3.74			
*Dermal density*				
Microneedling + conditioned medium	20.21 ± 4.99	0.13	2.46	0.13
Microneedling + normal saline	20.16 ± 5.08			

### Physicians and patient’s satisfaction

Physician assessment indicated a good and excellent response in 40% and 50% of patients, respectively, in the MN plus CM Group. No subjects showed little or no response. In contrast, 40% and 60% of patients had little and no response in MN plus NS group, according to physician evaluation. Notably, no patient achieved a good or excellent response in this group. The physician satisfaction rate was significantly higher in the MN + CM Group than in the MN + NS group (*p* = 0.001).

**Fig. 1:**
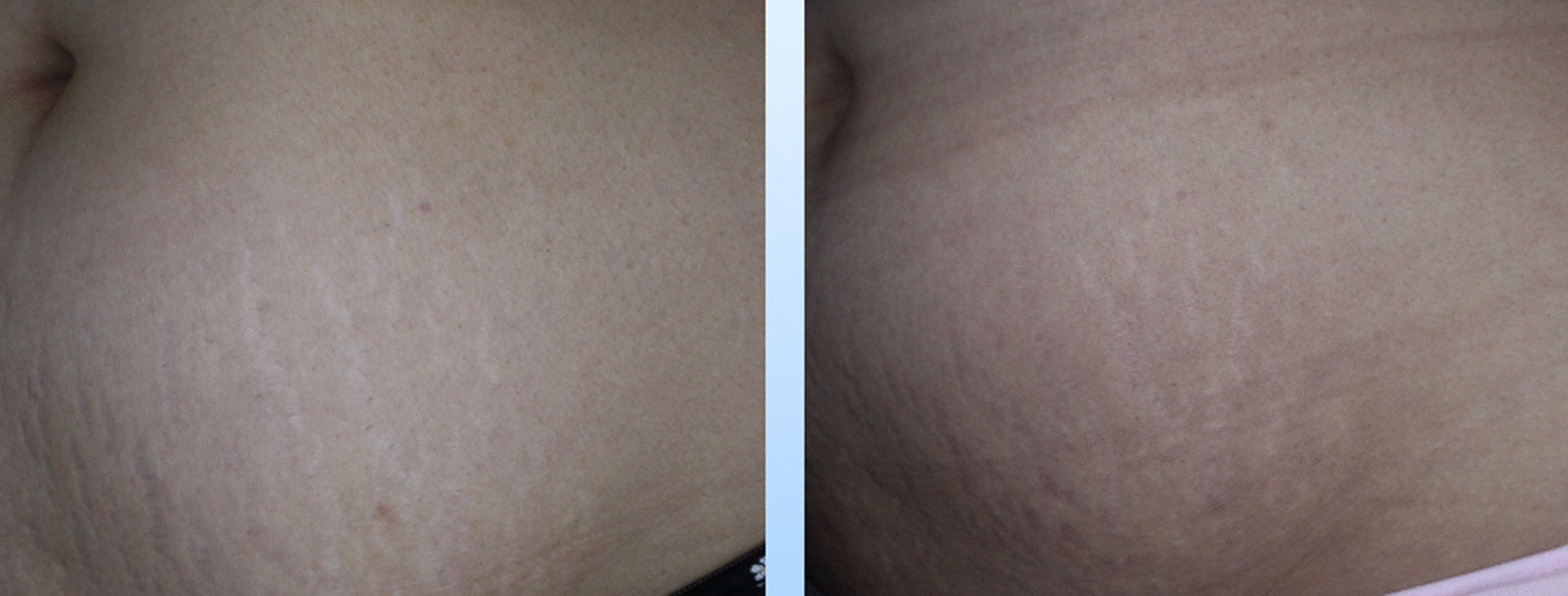
Needling and intradermal injection of conditioned medium, left: before, right: after

**Fig. 2:**
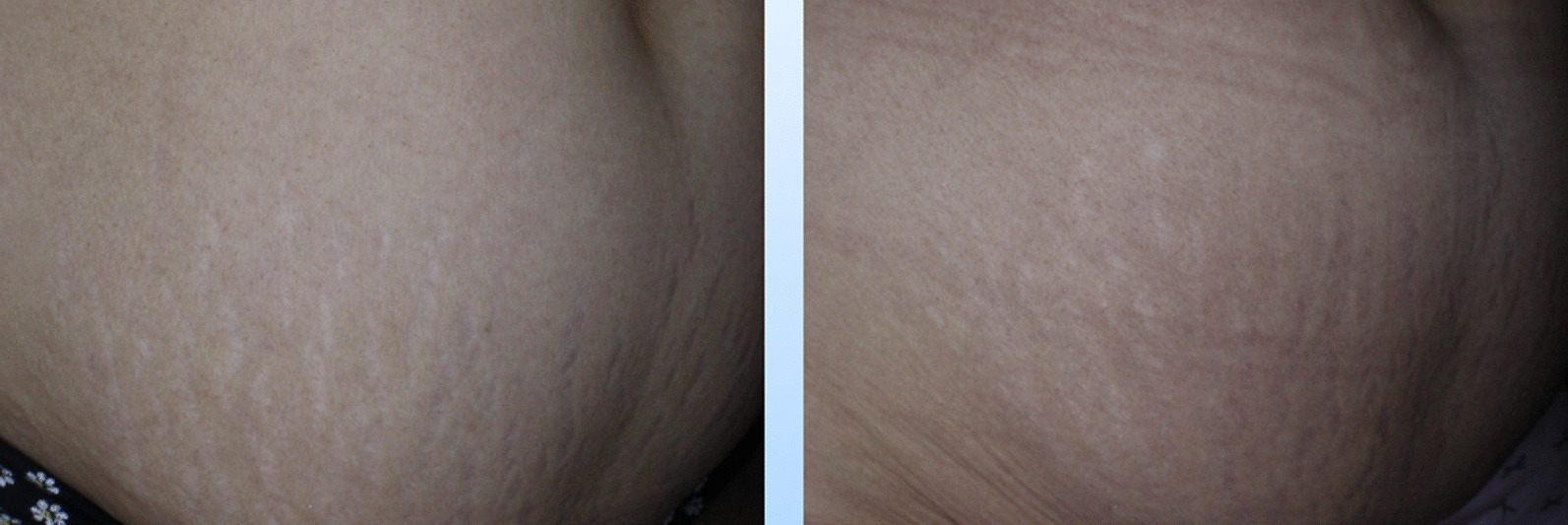
Needling and intradermal injection of normal saline, left: before, right: after

According to patient self-evaluation, 40% and 60% of patients in the MN + CM Group considered their lesion’s response as good and excellent, respectively three months after the intervention (Fig. 1). No one described the response to treatment as little or no change in the MN + CM Group. In contrast, 60% and 40% of patients in the MN + NS group considered the treatment as showing little and no change (Fig. 2). Consistent with the physician’s assessment, the patient satisfaction rate was significantly higher in the MN + CM Group compared to the MN + NS group (*p* = 0.001) (Table 3).

**Table 3:** Comparing the Physician’s and patient’s satisfaction levels between two groups

Satisfaction level	Microneedling + conditioned medium	Microneedling + normal saline	*p*
*Physician satisfaction*			
No response	0 (0.00)	6 (60.00)	0.001
Little	0 (0.00)	4 (40.00)	
Somewhat	1 (10.00)	0 (0.00)	
Good	4 (40.00)	0 (0.00)	
Excellent	5 (50.00)	0 (0.00)	
*Patient satisfaction*			
No response	0 (0.00)	4 (40.00)	0.001
Little	0 (0.00)	6 (60.00)	
Somewhat	0 (0.00)	0 (0.00)	
Good	4 (40.00)	0 (0.00)	
Excellent	6 (60.00)	0 (0.00)	

## Discussion

There has been increasing attention toward stem cell therapy in different fields, including dermatology, in recent years. Mesenchymal stem cells have been applied to various dermatologic conditions, especially in wound healing, scars, and rejuvenation [20]. Considering striae distensae as a kind of atrophic scar, there haven’t been any clinical trials conducted to evaluate the effectiveness of this trend regarding striae treatment.

This randomized clinical trial evaluated the efficacy of human umbilical cord mesenchymal stem cells plus microneedling compared to microneedling alone in striae distensae. Three 3-weekly sessions of dermal injection of conditioned media following microneedling resulted in notable patient and physician satisfaction compared to microneedling and normal saline.

Microneedling has been shown to be an effective treatment modality for striae distensae by increasing the production of elastin and collagen and enhancing epidermal thickness [11, 32, 33]. Several studies have demonstrated that microneedling is as effective as CO_2_ fractional laser in improving stretch marks, with less downtime, greater availability, and more cost-efficiency [30, 34, 35]. According to this study, microneedling plus normal saline did not lead to achieving satisfaction for either patients or physicians. Despite observing a trend in biometric parameters, only changes in dermal and complete thickness, as well as skin density, were defined as significant.

In 2023, Abbas et al. demonstrated that four monthly sessions of both microneedling and microneedling combined with Ascorbic Acid on 28 patients were safe and effective in treating striae distensae three months after completing the protocol [36]. Another study in 2019, conducted on 20 women with striae distensae, compared the effectiveness of non-ablative fractional laser (NAFL) to microneedling in five monthly sessions. Both modalities were effective based on clinical and histologic evaluation (*p* < 0.01), while laser had more downtime [34].

Our study revealed conflicting clinical findings, as both patient and physician assessments were unsatisfactory in control group. It seems that this contradiction might be due to the lower number of sessions, and further follow-ups were required, although some improvements were observed in biometric evaluation. Similar to resurfacing lasers, microneedling promotes collagen synthesis and dermal remodeling through dermal injury [29, 34, 37]. Concurrently, current study showed a notable increase in dermal and complete thickness and skin density using microneedling, which might be attributed to an increase in collagen bundles.

According to several systematic reviews, combination therapy is the most approved approach to treating striae distensae (SD) [8, 13]. The combination of microneedling with platelet-rich plasma (PRP) has been identified as a satisfactory approach to SD treatment [38]. PRP is considered a form of regenerative medicine containing numerous growth factors. According to Abdel-Motaleb et al.’s study in 2022, three monthly sessions of microneedling plus PRP on stretch marks in 40 patients resulted in notably higher patient and physician satisfaction and a marked increase in collagen and elastin fibers compared to microneedling alone [38]. Similarly, in our study, the patient and physician assessments were significantly higher in the MSCs plus MN group. Additionally, there was a significant enhancement in epidermal, dermal, and complete thickness, as well as density, in this group.

While combination therapy is generally considered the best approach to SD, contradictory results have been observed in different studies involving various modalities [39–41]. A review study conducted in 2021 to evaluate the effectiveness of the PRP method in treating striae distensae revealed that the published studies in this field did not provide strong evidence of the effectiveness of PRP [42].

Since the emergence of MSCs, many studies have applied the regenerative effects of these cells in a wide range of medical fields from cardiology to neurology. Several studies have implicated MSCs as a promising approach to treating scars, wounds, and skin regeneration in recent years [43, 44]. In this study, three monthly sessions of a combination of human Wharton’s jelly mesenchymal stem cells (hWJMSCs) with microneedling indicated statistically more patient and physician satisfaction with a significant increase in all skin ultrasound parameters (epidermal, dermal, and complete thickness and density). However, the cutometer parameters didn’t show any significant change. In 2023, Joo et al. [45] utilized three monthly sessions of non-ablative laser plus human stem cell-conditioned medium (HSCM) and compared it to laser alone on hypertrophic scars. The thickness changes of scars were significant in the combined group comparing to the control (*p* = 0.01). In 2021, Park et al. [46] compared the drug delivery of human stem cell-conditioned media (HSCM) following fractional CO_2_ laser to laser alone in the treatment of atrophic acne scars. Two months after a single session, scar volume was reduced by 23.5% in the combination approach compared to 15% in the control, which wasn’t statistically significant (*p* = 0.143). However, the volume of the skin pores was significantly reduced by 37.6% (T) versus 15.9% (C) (*p* = 0.006). In a similar study conducted in the same year [47], the efficacy of topical stem cell-conditioned medium (SC-CM), PRP, and NS following three monthly sessions of fractional CO_2_ laser resurfacing for atrophic acne scars was assessed. Despite a significant increase in dermal collagen in the SC-CM and PRP groups compared to the control, better clinical improvement was observed in PRP compared to SC-CM (*p* = 0.63). Similar to recently developed studies, some contrasting findings were observed regarding clinical and biometric comparison in the combination of MSCs with MN to MN plus NS in this trial. This indicates the need for further studies to clinically evaluate the effectiveness of stem cells in atrophic scars and striae distensae with more intervention sessions and longer follow-up. It could be suggested that due to the different pathological characteristics of atrophic scars from hypertrophic scars [48], more intervention sessions might be required to achieve statistically promoted outcomes, particularly in viscoelasticity and pure elasticity, since this promotion is somehow observed in our study and these studies. According to in vitro and animal studies, mesenchymal stem cells (MSCs) have an anti-fibrosis effect, the potential to reshape the microvascular structure, form complex hybrid systems, induce collagen synthesis, and remodel ECM, all of which are considered to play a role in the pathophysiology of SD [19, 49, 50]. Wharton’s jelly mesenchymal stem cells are considered a particularly better option due to their role in embryonic growth and development as well as tissue repair, compared to other cells [23]. Stromal vascular fraction (SVF), another regenerative modality, was employed in 2023 to evaluate its effectiveness in the treatment of burn scars [51]. Similar to our findings, three months of sessions involving a combination of SVF and fractional CO_2_ laser led to higher patient and physician satisfaction compared to laser alone. Additionally, epidermal thickness, complete density, and skin density sonography revealed a significant change in the combination group.

Combination of MSCs with MN (microneedling) has also been practiced in several studies, especially for rejuvenation with promising outcomes. In a randomized split-face study, five bi-weekly sessions of hUC-MSCs-CM (human umbilical cord mesenchymal stem cell-conditioned media) plus MN were employed on thirty subjects suffering from skin aging [52]. Compared to MN alone, the Combination group exhibited more assessor and patient satisfaction, as well as significant improvements in skin brightness parameters (reduced melanin index, ultraviolet spots, and brown spots) and skin texture (reduced wrinkles and pores, and increased skin elasticity) (*p* < 0.05) two weeks after the final session. Meanwhile, there were no obvious differences observed in skin hydration, trans-epidermal water loss, and the erythema index. In a separate split-face study in 2023, Adipose tissue stem cell-derived exosomes (ASCEs) with microneedling were applied in three sessions with 3-week intervals for skin rejuvenation and compared to MN alone [53]. Following 6 weeks from the last treatment, the clinical evaluation was much more remarkable in the combination group (*p* = 0.023). Significant improvement was observed in skin elasticity (*p* = 0.002), skin hydration (*p* = 0.37), skin pigmentation (*p* = 0.044), and greater density of collagen and elastic fibers in the combination group. Another study showed that five bi-weekly sessions of skin needling and topical application of amniotic fluid mesenchymal stem cell-derived conditioned media (AF-MSC-CM) would significantly result in improving skin (*p* < 0.001) and better remodeling of the dermal structures [54].

Up to date, we haven’t discovered any clinical trials evaluating the effectiveness of human mesenchymal stem cells in stretch marks and comparing it in combination with microneedling (MN), one of the common traditional methods, to MN alone. Our study represented the first clinical trial evaluating the effectiveness of mesenchymal stem cells for the treatment of striae.

Supporting previous clinical trials in scars, our findings suggest that MSCs might be a promising option. The limitations of the current study include a low number of sessions, a short follow-up, and a small sample size, which may result in non-significant biometric differences between the two groups.

## Conclusion

Based on the results of the present study, the combination of CM with MN shows promising outcomes in the treatment of SD compared to microneedling alone, as indicated by patient and physician evaluations. However, regarding biometric assessment, this combination was not superior to the control group. Further studies with increased intervention sessions, longer follow-up periods, and larger sample sizes are suggested.

## Data Availability

The data supporting the results of this study are available from the corresponding author.
